# Blockchain-Based Caching Architecture for DApp Data Security and Delivery

**DOI:** 10.3390/s24144559

**Published:** 2024-07-14

**Authors:** Daun Kim, Sejin Park

**Affiliations:** Department of Computer Engineering, Keimyung University, Daegu 1095, Republic of Korea; kimdaun@stu.kmu.ac.kr

**Keywords:** blockchain, decentralized applications, data integrity, distributed system, user privacy, cache coherence

## Abstract

Decentralized applications (DApps) built on blockchain technology offer a promising solution to issues caused by centralization. However, traditional DApps leveraging off-chain storage face performance challenges due to factors such as storage location, network speed, and hardware conditions. For example, decentralized storage solutions such as IPFS suffer from diminished download performance due to I/O constraints influenced by data access patterns. Aiming to enhance the Quality of Service (QoS) in DApps built on blockchain technology, this paper proposes a blockchain node-based distributed caching architecture that guarantees real-time responsiveness for users. The proposed architecture ensures data integrity and user data ownership through blockchain while maintaining cache data consistency through local blockchain data. By implementing local cache clusters on blockchain nodes, our system achieves rapid response times. Additionally, attribute-based encryption is applied to stored content, enabling secure content sharing and access control, which prevents data leakage and unauthorized access in unreliable off-chain storage environments. Comparative analysis shows that our proposed system achieves a reduction in request processing latency of over 89% compared to existing off-chain solutions, maintaining cache data consistency and achieving response times within 65 ms. This demonstrates the model’s effectiveness in providing secure and high-performance DApp solutions.

## 1. Introduction

In traditional centralized systems, users relinquish control over their data to centralized authorities, raising concerns about data misuse and unauthorized access. Furthermore, centralized architectures are susceptible to single points of failure, making them vulnerable to external attacks such as hacking and DDoS attacks as well as performance degradation due to high loads. The rise of decentralized services has revolutionized various industries, offering a compelling alternative to traditional centralized models [1].

To address these inherent issues in centralized systems, decentralized solutions based on blockchain technology [2] have been introduced. Blockchain relies on immutable distributed ledger technology, enabling trustworthy data management without central authorities. This characteristic eliminates single points of failure, enhancing security and reliability. The most important factor enabling decentralized applications is smart contract technology [3] operating on the blockchain. Smart contracts provide automated contract execution without third-party intervention, as contract terms are encoded into code and executed automatically on the blockchain network’s virtual machine. This capability enables secure and reliable operations across various domains, such as the Internet of Things (IoT), healthcare systems, digital rights management, financial systems, and real estate within the system [4].

### 1.1. Motivation

This section discusses the limitations present in existing research and provides an explanation for the necessity and motivation behind this research.

As blockchain technology continues to permeate diverse industrial sectors, the demand for real-time functionality is becoming increasingly evident. However, the inherent scalability issues in blockchain technology, particularly around storage, continue to pose challenges when utilizing blockchain in decentralized services. In decentralized applications, data operations rely only on smart contract functions, resulting in slow throughput and large resource requirements [5]. Smart contracts exist on the blockchain, making both their code and data publicly accessible. Consequently, malicious actors can exploit the code, and there are concerns about privacy infringement when sensitive information is exposed to anyone. Moreover, smart contracts face challenges in resolving performance issues in resource-constrained execution environments [6]. Therefore, addressing these issues requires approaches that consider security and privacy concerns while minimizing or efficiently managing resource utilization. To address these concerns, off-chain storage solutions have gained attention; data generated by actual services can be stored in external cloud storage, while only the hash values of the original data are maintained for integrity [7,8]. Utilizing shared cloud storage among devices for data operations can save resources required for smart contract execution. Thus, a hybrid approach utilizing both off-chain and on-chain storage offers a promising solution by combining real-time interaction with the advantages of blockchain [9].

However, the cloud services used for off-chain storage are typically limited to providing average throughput via large-scale expansion. End-to-end latency during cloud search operations mostly arises from communication delays. Traditional cloud-based infrastructure can lead to increased latency, particularly for geographically dispersed users. This latency can significantly degrade user experience. Additionally, decentralized storage solutions such as IPFS may suffer from diminished download performance due to I/O constraints influenced by data access patterns [10]. In summary, existing off-chain storage solutions such as IPFS and cloud storage impose performance limitations on real-time requirements. When applied to actual decentralized applications, the system’s performance is heavily reliant on the off-chain storage location, network speed, and hardware conditions, leading to reduced Quality of Service (QoS) due to off-chain storage latency [11]. Digital services are highly susceptible to latency issues, and even slight delays can significantly degrade user experience, leading to revenue loss [12].

Therefore, response latency is a critical factor to consider when designing decentralized services. Our research aims to address the latency issues inherent in existing decentralized application solutions by proposing a blockchain-based caching architecture. In order for decentralized services to be universally applicable across various fields, it is essential to consider the real-time capabilities provided by traditional centralized structures. We propose an architecture that utilizes a blockchain-based cache cluster to store and retrieve decentralized service data. By enabling direct queries to cache nodes holding the content, our approach eliminates the need for cloud requests, thereby minimizing latency and enhancing overall performance. Additionally, our system leverages off-chain storage on blockchain nodes to cache data, effectively reducing traffic for off-chain storage requests and ensuring data integrity, ultimately providing users with superior QoS. In addition, we adopt asset-based groups to enable secure sharing of content stored on the blockchain.

### 1.2. Contribution

In this section, the contributions of the proposed research are delineated. The proposed architecture contributes to ensuring the reliability of nodes and consistency of cached data through a blockchain node-based off-chain storage content caching system. Additionally, it supports granular access control for content sharing using attribute-based encryption, contributing to data protection in untrusted cloud storage and distributed caching system environments. Lastly, by utilizing blockchain nodes as cache nodes and forming regional cache clusters, it optimizes request performance, reduces latency, and enhances system responsiveness.

The rest of this paper is structured as follows: Section 2 delves into research pertinent to blockchain-based caching and access control mechanisms; Section 3 elucidates the background technology that underpins our proposed system; Section 4 details the specific design of the proposed system; Section 5 discusses the experiments conducted and the evaluations performed to assess the efficacy of our approach; finally, Section 6 presents the conclusions.

## 2. Related Work

In this section, we describe research on caching techniques and access control research utilizing blockchain. Following this, a comparative analysis contrasts the distinguishing features between our proposed approach and existing systems in this field.

Research on systems that introduce blockchain technology to address the data security issues of traditional centralized systems is gaining attention. Alrebdi et al. [13] incorporated blockchain into electronic medical systems to ensure decentralization, security, anonymity, immutability, and tamper-resistance. The system provides storage, verification, and retrieval through smart contracts and employs IPFS and cloud computing to store patient data. Zhang et al. [14] proposed a blockchain-based privacy-preserving e-health system to solve the security issues of cloud-based Electronic Health Records (EHRs). By introducing pairing-based encryption, they created tamper-proof EHR managed through contracts.

### 2.1. Caching Research for Distributed Environments

In this section, we introduce caching in distributed environments, discuss related works, and compare each work, with a focus on caching architectures. The importance of caching research applicable to distributed environments, such as edge computing to improve the performance of central servers or cloud environments, has been emphasized in several works. Yamanaka et al. [15] proposed a user-centric caching mechanism within an off-chain storage network based on information-centric networking, which overcomes the limitations of existing off-chain solutions by providing efficient data transmission and high access performance. Bai et al. [16] proposed a pre-caching strategy and a distributed caching technique in a blockchain-based mobile edge computing environment. Performance improvements were achieved through caching optimization utilizing node utilities, and a penalty incentive mechanism for caching operations was introduced to increase system utility and stability in a distributed environment. However, offline proof between the data provider and the cache node causes oracle problems and does not guarantee the stability of the system utilizing smart contracts. Guo et al. [17] proposed a caching optimization model and data storage method that utilizes blockchain in an edge environment to cache data while meeting user requirements and increasing the cache hit rate by predicting the cache contents. Zhang et al. [18] proposed a three-layer caching mechanism that adds a cache node layer through a smart contract, allowing users to efficiently obtain content through a three-layer cache in a large-scale network environment while providing increased response speed and reduced network overhead. Large files are transformed into a Merkle tree structure, encrypted, and stored using an IPFS system, then a Third-Party Auditor (TPA) performs integrity verification. Heo et al. [19] designed Multi-Level Distributed Caching (MLDC), a multi-level distributed caching strategy based on node availability for blockchain storage optimization. It reduces blockchain storage requirements by proposing that one node caches only those data that it needs, rather than holding the data of the entire blockchain. Wang et al. [20] proposed a distributed framework for dictionary caching in blockchain-based hierarchical wireless networks. An autonomous content caching transaction is constructed using smart contracts, and an incentive mechanism is used to activate cache helpers by combining blockchain and game theory perspectives. Freedman et al. [21] designed CoralCDN, a web content distribution network that distributes web content by leveraging the bandwidth of volunteers to reduce the burden on origin servers. Saturn [22] implements a content delivery network that accelerates content delivery by designing L1 nodes that cache data stored in the InterPlanetary File System (IPFS), promoting content delivery network activation through incentive mechanisms. Table 1 presents a summary and comparison of the proposed studies.

### 2.2. Research on Blockchain-Based Access Control Mechanisms for Security

This section introduces research related to blockchain-based access control mechanisms that ensure system stability.

In security, access control is a critical component that restricts unauthorized users from accessing resources or accessing resources in unauthorized ways. Preventing such access is especially crucial in distributed environments. In distributed environments, access control not only prevents unauthorized access, it also fosters trust among users and stakeholders by ensuring that sensitive information remains secure across diverse network nodes and interconnected systems. Research on solutions enabling management of private keys and performing access control without third-party intervention in distributed environments is actively being pursued.

Horvath et al. [23] proposed methods for making attribute-based encryption more suitable for access control to data stored in the cloud. Sukhodolskiy et al. [24] designed a prototype of a multi-user system capable of controlling access to datasets stored in untrusted cloud environments using dynamic attribute-based encryption policies. Sharma et al. [25] proposed a blockchain-based framework with a CP-ABE algorithm to provide access control and user revocation methods for cloud storage systems. Wang et al. [26] proposed a cloud storage framework combining the Ethereum blockchain and CP-ABE. Li et al. [27] proposed a fog computing-based blockchain-supported access control system to address privacy issues in a Smart Healthcare System (SHS). This system adopts BC-assisted multi-authority attribute-based encryption (MA-ABE) with keyword search to implement fine-grained access control. Because the approach is not suitable for resource-constrained IoT environments, most computational tasks are outsourced to fog nodes. Alharbi [28] enhanced centralized Cybersecurity Information Sharing (CIS) using the Access Control-Enabled Blockchain (ACE-BC) framework to bolster data security. This framework restricts unauthorized access using attribute-based encryption technology. Yang et al. [29] proposed a blockchain-based access control framework called AuthPrivacyChain with enhanced cloud security features with the aim of protecting personal information. This framework designs access control, authorization, and revocation processes to prevent illegal resource access by hackers and cloud administrators. Han et al. [30] proposed an auditable access control model based on attribute-based access control (ABAC) to overcome security support issues in access control processes due to the limited performance of IoT devices. This model stores request logs, response logs, and access logs on the blockchain to manage access control policies and enable auditing.

Previous studies have adopted blockchain in caching technology to evaluate the integrity and usefulness of caching node roles and introduce incentive mechanisms. However, this paper is different in that it proposes a blockchain-based caching technique that focuses on ensuring the consistency of caching content and protecting content integrity in Web3.0 application environments. Through this architecture, performance improvement of blockchain-based distributed applications is achieved.

### 2.3. Research Gap Analysis

In this section, we discuss the differences between related previous research and the proposed research. Table 2 illustrates the distinctions between the described studies and the proposed research.

Research not focusing on system architecture is excluded from the comparison with the proposed system. The comparison criteria include decentralization, caching systems, preventing cache poisoning, data integrity, and access control. The first category assesses the degree of decentralization and the extent to which single points of failure are avoided; a “low” rating indicates the presence of central points of failure, a “middle” rating indicates the integration of blockchain networks with cloud storage, and a “high” rating indicates an architecture capable of integrating blockchain networks with distributed storage. Cache system indicates whether a distributed caching system utilizing edge nodes is implemented to delegate original data to other nodes for performance improvement. Preventing cache poisoning examines whether the system avoids cache poisoning risks among the edge nodes utilizing each cache system. Data integrity evaluates whether the system ensures the integrity of the original data stored in external storage. Finally, access control describes whether the system supports access control for the security of shared data. The proposed system can achieve a fully decentralized shared system by adopting distributed storage as an off-chain storage layer and utilizing blockchain nodes to form a distributed cache cluster. Additionally, it improves performance through a caching system and prevents cache poisoning threats by verifying the integrity of updated data and identifying modified data via the local blockchain ledger. The integrity of the data is ensured by storing the hash value of the original data on-chain. Furthermore, the proposed system supports access control for data stored in untrusted storage using attribute-based encryption algorithms. In contrast, existing research does not address the prevention of cache data corruption or access control of the data transmitted within the system. By considering all these aspects, we propose a reliable blockchain-based cache system that completely prevents data leakage and tampering.

## 3. Background

In this section, we provide explanations of the notation used in the paper and discuss two key background technologies, namely, blockchain and Ciphertext Policy–Attribute-Based Encryption (CP-ABE).

### 3.1. Blockchain-Based Decentralized Apps

In this section, we explain the characteristics of blockchain technology and its application in decentralized applications.

Blockchain, first introduced as the underlying technology of Bitcoin, has developed into a powerful and secure distributed ledger system that can be applied to a variety of fields beyond digital currency. A blockchain is a distributed immutable ledger that records transactions transparently. Each block in the blockchain contains a list of transactions and the hash of the previous block, creating a chain of linked blocks. Unlike centralized databases, blockchains operate network-wide, with each node maintaining a copy of the entire ledger. This makes data tampering virtually impossible, as changing a single block requires changing all subsequent blocks in the network. Blockchain technology uses cryptographic methods such as hashing and digital signatures to secure data and verify the authenticity of transactions without intermediaries.

Decentralized Applications (DApps) are applications that run on a blockchain, providing a trusted environment without the need for intermediaries or central authorities. DApps have spread across various fields, including finance, healthcare, and social networking. DApps leverage the security and decentralization of blockchain technology to provide features such as improved privacy and user control over data. Smart contracts, self-executing pieces of code deployed on a blockchain, are a core component of DApps. Contracts are distributed across the blockchain network and executed by multiple nodes, and all nodes equally verify the execution results of the contract. This means that unlike centralized systems, the entire network verifies and executes contracts without a single administrator or intermediary. These distributed characteristics increase reliability and transparency while preventing forgery, falsification, and data fraud.

### 3.2. Ciphertext Policy–Attribute-Based Encryption

This section provides a detailed explanation of the features and functions of attribute-based encryption for access control.

Public key encryption [31] allows for bidirectional communication in which data are encrypted for secure transmission to a single recipient by utilizing the recipient’s public key over a public channel. However, this bidirectional model presents limitations regarding encryption for multiple users, including unknown or potential users. To achieve scalable and secure data sharing in untrusted distributed environments, Ciphertext Policy–Attribute-Based Encryption (CP-ABE) [32] enables fine-grained access control over data. CP-ABE expresses access policies as attributes and encrypts data based on these attributes, determining decryption based on the match between user and data attributes. The encryption technique used for our access control is based on the CP-ABE method optimized for cloud computing, as proposed by Horvath [23]. The functionality of the proposed CP-ABE is outlined in Table 3.

Initially, the system is identified through circuit initialization, setting up global system parameters (GP). The configCA function establishes the public key pair (SK∗,PK∗) to configure the Certification Authority (CA). The CA plays a role in assigning attributes in the system environment. Using genUserPrivKey, the CA generates individual private keys (*K*_GID_) for users participating in the system by utilizing its own private key (SK). Each CA can create public key pairs (SK,PK) for attributes through genAttrKey. Subsequently, users receive attribute private keys (*K*_*i*_) and user identification public keys (*K*_GID_) using the attribute public key, their global ID (GID), and the attribute index (*i*). By generating individual identification keys alongside attribute private keys, CP-ABE prevents situations in which multiple users collude to decrypt ciphertexts encrypted with multiple attributes. Encryption involves encrypting content *M* using the GP, attribute policy *P*, a set of PKs, PK*, and a revoked user list (RL). To decrypt ciphertexts, users require their attribute private key set ({*K*_*i*_, *K*_GID_}) and user identification private key (K*_GID_). Trustworthy CAs issue attribute keys, allowing users to decrypt ciphertexts using attribute private keys obtained from CAs through the utilized attribute-based public keys. Attribute-based encryption utilizes various operators to classify users with diverse attributes, allowing only users possessing designated attributes to access data. This can be utilized to provide subsequent access for users who join later to ciphertexts encrypted with attribute keys, as issuance of the user’s attribute private key enables granular access for multiple users using a single ciphertext.

Table 4 presents the frequently used notation in this paper.

## 4. System Design

In this section, we propose a blockchain-based caching architecture and explain the caching system processes. Additionally, we define the specific processes of the key components constituting the system and describe the security systems that configure the access control.

### 4.1. Blockchain-Based Caching Architecture

This section provides an overall overview of the blockchain-based caching architecture, detailing its structure and the roles of each layer within the architecture. Furthermore, it includes descriptions of both the conductor and the cache node supporting the caching system.

**Figure 1:**
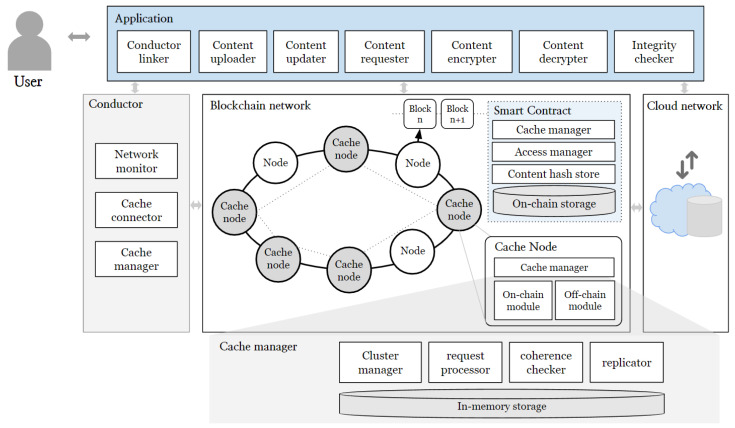
Overview of blockchain based caching architecture.

The proposed system assumes that users interact with the service system through the application interface and that user data produced within the service are stored in cloud storage. Figure 1 illustrates the overall structure of the system operating in this environment. The system is broadly composed of three layers: the Application layer, the Blockchain layer, and the Cloud Network layer.

Users can upload content through the system and download desired content. Additionally, they are empowered to modify their uploaded data, thereby realizing data control authority. Comprehensively, the application is divided into seven main modules, each performing a specific function within the system. These modules are as follows:

- **Conductor linker**: Handles essential connections with the conductor, whose role is elaborated later.
- **Content uploader**: Users utilize this module to upload their desired content. The content is uploaded to the cloud network, and its hash value is also uploaded to the Blockchain Network (BCN) to ensure data integrity.
- **Content updater**: Allows users to update their data stored in cloud storage. To ensure the integrity of the updated content, the update must be reflected through the BCN.
- **Content requester**: Users can request data stored in the cloud using this module. Using the content’s ID as the key, a request is sent to Cache Nodes ($N_{cache}$) within the BCN. The $N_{cache}$ then delivers the requested data to the user.
- **Content encryption and decryption**: Supports content encryption and decryption on a single user device while protecting data stored in external storage by ensuring that only authorized users can decrypt and access the content.
- **Integrity checker**: This module verifies the integrity of received data through the blockchain. It enables the verification of the reliability of external storage and prevents incorrect data transmission by malicious $N_{cache}$.

The Blockchain Network (BCN) consists of a set of blockchain nodes (*N*) and multiple cache nodes ($N_{cache}$). All network nodes, including users and the blockchain, must have accounts within the blockchain. One of the cache nodes serves as a conductor node ($N_{cdt}$).

To enhance performance on the public BCN, multiple $N_{caches}$ are assumed to be available. The Cache Manager module, operated by $N_{caches}$, performs caching. Smart contracts for managing the system and $N_{caches}$ are deployed. These contracts include:

- **Cache manager contract**: Maintains and manages a list of $N_{caches}$ to ensure reliability. One node from $N_{cache}$ is selected as the $N_{cdt}$, which manages node departures and controls malicious nodes.
- **Access manager contract**: Handles access control for each user. It maintains a list of Global IDs (GIDs) of users within the BCN and maps the addresses of each access control contract. Users can assign properties to request users and set access ranges for shared content using attribute-based encryption.
- **Content hash store contract**: Stores the hash values of content kept in off-chain storage and ensures the integrity of off-chain content. Data owners can sign updated data and update hash values when the content in off-chain storage changes.

In cloud networks, off-chain storage is provided to handle the storage of original data uploaded by users onto the system. The integrity of stored data is verified through its hash values managed on the blockchain, ensuring storage reliability. Moreover, all data are encrypted before storage, ensuring safety from leaks. This layer can employ distributed file storage systems or centralized servers, enabling fully decentralized system operation. Ultimately, the system is designed with a caching architecture comprising blockchain network layers, cloud network layers, applications, and cache clusters, ensuring reliability and performance improvement.

#### 4.1.1. Location-Based Cache Clustering with Conductor

This section provides a detailed explanation of the specific roles performed by the conductor in supporting the caching system.

**Figure 2:**
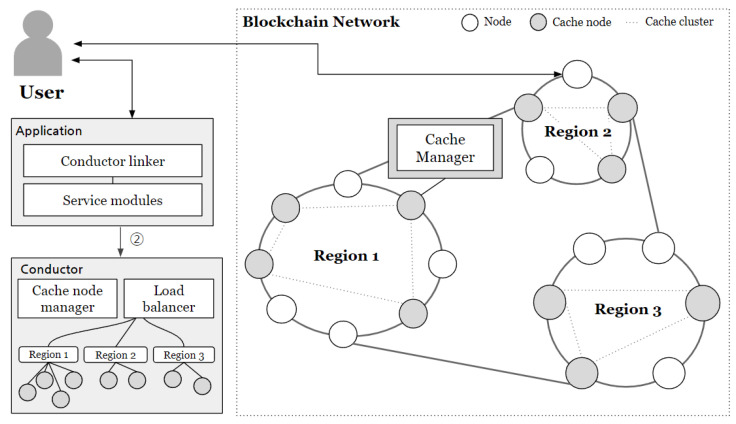
Cache cluster formation by the conductor.

The conductor’s role is to select appropriate cache nodes that deliver content when users first request it. To achieve this, the conductor ($N_{cdt}$) maintains IP and location details for each cache node ($N_{cache}$) in order to form cache clusters per region. The cache nodes configure these clusters by identifying nearby ones through the conductor, and also manage routing information. This setup allows physically adjacent nodes to form cache clusters, allowing for multiple clusters on a single Blockchain Network (BCN). Figure 2 illustrates how the conductor organizes cache clusters by region.

The clusters are operated by $N_{caches}$, and aim to reduce latency by processing requests from nearby users. In addition, they ensure cached data coherence and replicate data for availability, ensuring efficient and stable cache clusters.

#### 4.1.2. Cache Node Architecture

**Figure 3:**
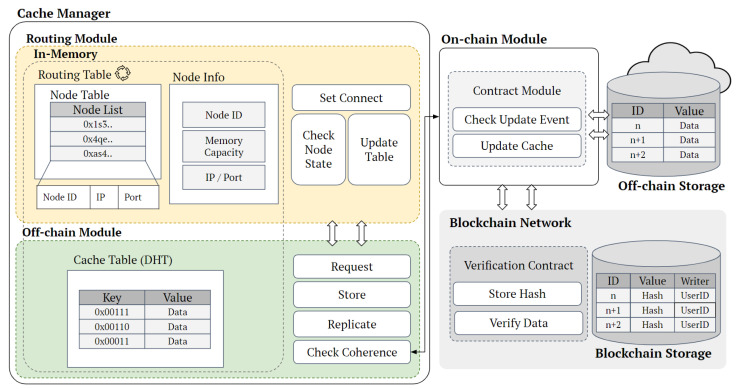
Cache node architecture combining blockchain and off-chain storage.

This section includes a detailed explanation of the components and operations of the Cache Manager module, which runs on cache nodes within the blockchain network. The components of the cache nodes are depicted in Figure 3. The cache manager module includes a Routing Module and an Off-Chain Module that work in tandem to manage cache node information and cache data, respectively.

The Routing Module maintains a Node Table with Node IDs, IP addresses, and port numbers alongside Node Info detailing memory capacity. The Set Connect function facilitates the establishment of connections within the cluster, while the Check Node State and Update Table functions are responsible for continuously monitoring the status of nodes and updating the routing table to reflect any changes, ensuring that it remains current and accurate.

The Off-Chain Module utilizes a Distributed Hash Table (DHT) to manage cache data. It stores keys and encrypted data values in memory, ensuring quick access and efficient retrieval of requested data. Key functions within this module include:

- Request: Initiates requests for specific data stored within the DHT.
- Store: Stores data into the DHT, ensuring redundancy and availability across nodes.
- Replicate: Copies data across multiple nodes to enhance resilience and availability.
- Check Coherence: Maintains data coherence by verifying consistency and ensuring update events propagate across all nodes.

These functions collectively manage data requests, storage, replication, and coherence within the cache, ensuring data integrity and efficient retrieval.

Additionally, the On-Chain Module integrates with the blockchain to enhance data integrity and consistency. The Contract Module monitors update events from contracts deployed on the blockchain, and processes such as Check Update Event and Update Cache verify changes to the data and reload updated data from off-chain storage, ensuring the cache table is synchronized with the latest data. The Blockchain Network component further supports data verification and integrity through the Verification Contract, which stores hashes of data and verifies their integrity by comparing with the hashes stored on the blockchain. Off-chain storage serves as an external data storage solution, efficiently managing large datasets. Through these comprehensive modules, the Cache Manager establishes a robust system that enhances content delivery performance by utilizing both on-chain and off-chain storage solutions, ensuring rapid data access, high availability, and data consistency across the network.

### 4.2. Process of the Caching System

**Figure 4:**
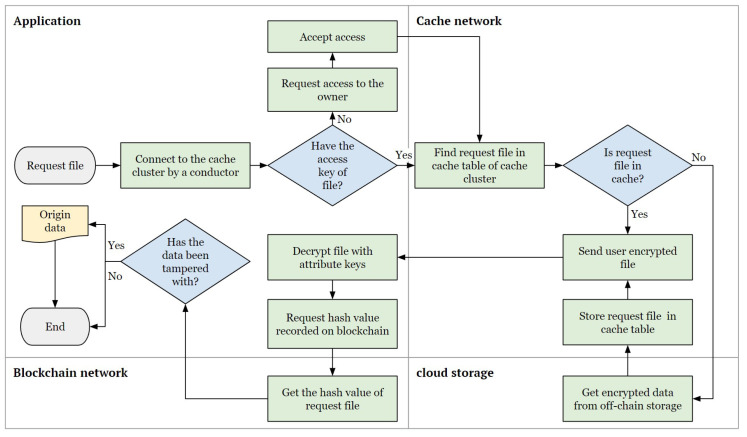
Caching system workflow.

This section provides a detailed explanation of how the proposed architecture operates. The caching process in the proposed system activates when data are requested. Thus, when a data request is made, the system caches the requested data and returns the data if they were already cached. Figure 4 shows a flowchart of how the file request process operates. The sequence of the request flow is as follows:

1. **File request to cache node**: The process begins with the user requesting a file through the application. The user is connected to the nearest cache cluster by the conductor. After connection, the user can send their request to the cache node with the fastest response time in the cache cluster. Before sending the request, the user must verify that they have the attribute keys to access the file. If the user has the attribute key for the file, they send the request to the cache node. If not, the user must request access from the file owner to obtain the attribute key. Details regarding key generation and issuance are explained in Section 4.4. After control approval, the file request is sent to the cache network.
2. **File search and delivery**: The cache node that received the request searches for the node within the cache cluster that caches the requested file based on the file ID. If the file is found in the cache cluster, then it is delivered to the user. If the file is not found in the cache cluster, then the original file is retrieved from the off-chain storage and cached. The cached file is then delivered to the requester.
3. **File integrity verification**: After receiving the file, the user decrypts it and retrieves the hash value corresponding to the file ID from the blockchain network. The user verifies whether the received hash value matches the decrypted data. If the hash values do not match, the data are considered tampered with, and the request process terminates. If the data are not tampered with, the integrity of the data is ensured and the original data are output to the user, concluding the process. If the user does not receive the original data and the process terminates, it is possible that the cache node may have maliciously tampered with the data. In such cases, the user must verify the reliability of the cache node by directly retrieving the data from the off-chain storage.

### 4.3. Main Component

This section elaborates the detailed requirements and process of the main functionalities: connection of the cache nodes via the conductor, content uploading and cache integrity verification, content requesting, content updating, and maintaining content coherence.

#### 4.3.1. Connection of Cache Nodes via Conductor

**Figure 5:**
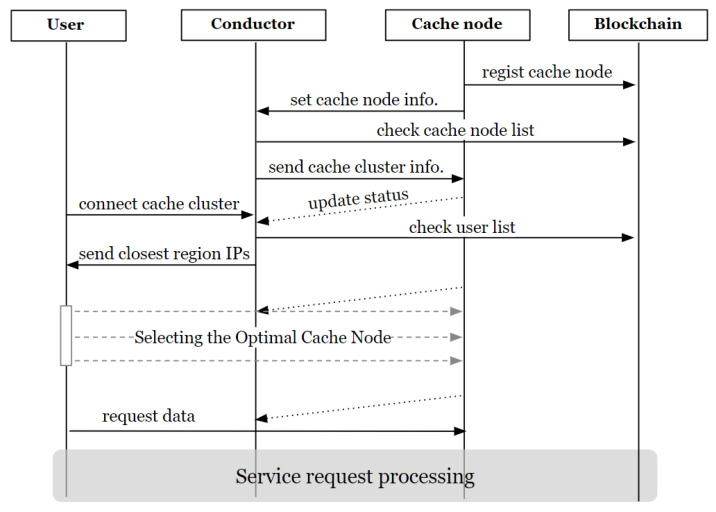
Cache node connection flow diagram.

Figure 5 illustrates the flow diagram of the process.

$N_{caches}$ register themselves as cache nodes on the blockchain. Smart contracts proceed with registration when the registration GID matches the $N_{cache}$ signing the transaction. Subsequently, the cache node proceeds with initial setup to join the cluster through the $N_{cdt}$. At this point, the $N_{cache}$ transmits its public key (PK) and the signed message $Sig(N_{cache}$_(ip, port)_) encrypted with $N_{cdt}$’s public key to the $N_{cdt}$. The $N_{cdt}$ verifies the received $N_{cache}$’s PK against the registered entries in $SC_{cacheManager}$ and authenticates the signed $N_{cache}$_(ip, port)_ to confirm the node’s authenticity. It records the $N_{cache}$’s information and timestamp based on its IP address in the corresponding region (defined as countries in the system).

Because each cluster is regionally distinguished, the $N_{cdt}$ forwards the recorded cluster information to the $N_{cache}$, allowing it to join the appropriate regional cluster. Subsequently, the $N_{cdt}$ monitors the $N_{cache}$’s status and periodically updates the region table.

For user connection requests, users initially transmit their public key $U_{\mathrm{pub}}$ and the signed message $Sig(U_{\mathrm{pub},\mathrm{ip},\mathrm{port}})$ to $N_{cdt}$. $N_{cdt}$ verifies the user’s registration status and provides a set of $N_{caches}\left(ip\right)$ closest to the user’s $U_{ip}$ based on the nearest region. The Haversine formula is used to calculate the distance between the user’s city coordinates and the cluster capital coordinates to determine the proximity.

Users send arbitrary requests to the received set of $N_{caches}$ and select the $N_{cache}$ with the fastest response time. The selected $N_{cache}$ then serves content delivery to the user. This process, facilitated through $N_{cdt}$, ensures the selection of the nearest and fastest $N_{cache}$, thereby minimizing the transmission delay.

#### 4.3.2. Content Uploading and Cache Integrity Verification

This section provides a detailed explanation of the requirements and processes involved in connecting cache nodes through the conductor. Users encrypt the content they intend to upload and register it in off-chain storage. To prove the integrity and ownership of the content, users record the hash value of the uploaded content through SC_hashStore_. Subsequently, other users can decrypt the content and verify its hash value using the verify function of SC_hashStore_. Algorithm 1 shows the operation process. This process ensures the trustworthiness of the content regardless of the trust level of the cache nodes or off-chain storage.
**Algorithm 1:** Content Upload and Cache Integrity Verification
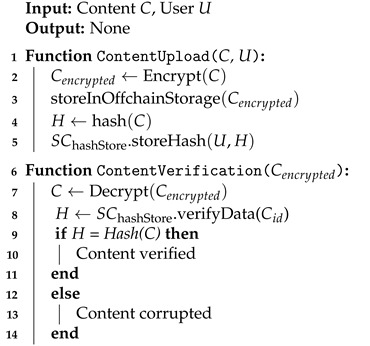


#### 4.3.3. Content Request

When a user requests content from the service $N_{cache}$ using the content ID, the $N_{cache}$ receiving the cache request initiates a search in the cache cluster to determine whether the requested content exists. This search involves querying nodes within the cluster based on the hash value of the content ID in order to identify the node holding the content. If the search reveals that the content is cached in the cluster, the $N_{cache}$ retrieves and returns the content. However, due to the limited storage capacity of each $N_{cache}$, cache misses may occur. In case of a cache miss, the $N_{cache}$ loads the data directly from off-chain storage corresponding to the content ID for delivery to the requester. Subsequently, the requested data are stored in the cache table and requests are made to cache the data in two randomly chosen nodes, thereby ensuring the recent data are maintained in the cache and enhancing the availability of cache data. If the cache table becomes full, the Least Recently Used (LRU) policy is employed to replace data. Upon receiving a response to the request, the requester measures the response time. If the actual delay exceeds the processing time required by the $N_{cache}$, then the requester can choose a new $N_{cache}$. Moreover, if no response is received within a specified time frame, then a transaction is sent to the BCN to remove the designated $N_{cache}$ from the list maintained by $SC_{cacheManager}$. Algorithm 2 shows the operation process. This process enables the monitoring of $N_{cache}$ performance and detects any abnormal behavior in an unreliable distributed environment.
**Algorithm 2:** Content Download Request Process
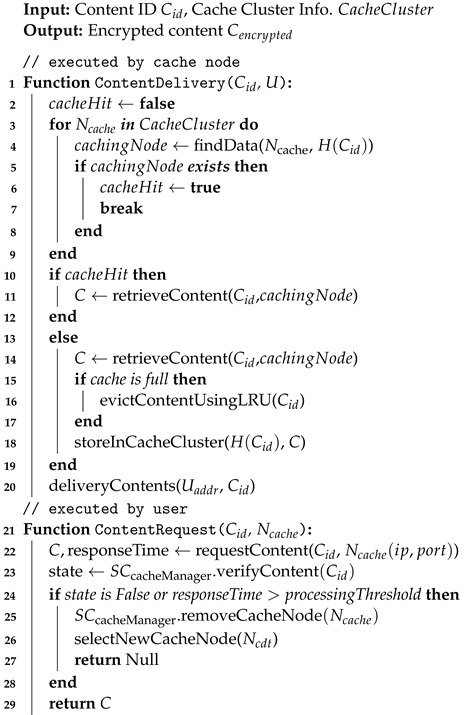


#### 4.3.4. Content Update and Coherence Maintenance

Ensuring coherence in a distributed caching system is essential. A process is required to maintain coherence between off-chain data and cached data. Each $N_{cache}$ holds a ledger of the BCN locally, enabling it to ensure coherence of the cached data by comparing the data with its own maintained ledger data. However, delivering the content to users after checking each request for coherence results in additional overhead and performance degradation. Because content updates by users are optional, performing coherence checks for every request is unnecessary. Therefore, we design the system utilizing smart contract events to incur coherence-related overhead only when update events occur.

Initially, users update the content corresponding to the Content ID ($C_{id}$) to the modified content and request storage in the $SC_{hashStore}$ by signing the transaction. After verifying the transaction, SChashStore triggers an update event upon confirming that the user who signed the transaction requested a change in the hash value corresponding to the Cid they authored. As each *N* executes transactions within its respective SC virtual machine, every *N* executing the transaction can immediately detect the occurring event. Consequently, $N_{caches}$ listening to such events promptly detect the update and retrieve the updated data from off-chain storage if there is correspondence with their own cached data. Algorithm 3 shows the corresponding operation process. This process ensures rapid and efficient maintenance of coherence.
**Algorithm 3:** Content Update and Coherence Maintenance Process
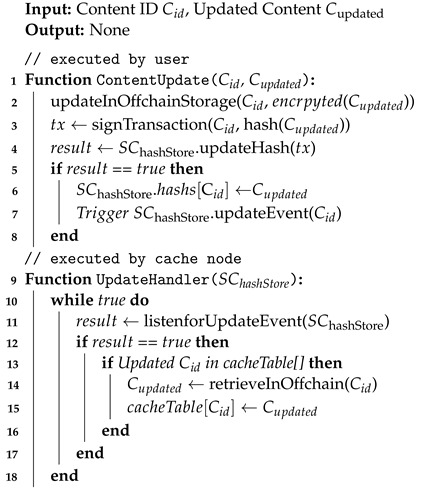


### 4.4. Distributed Access Control from Off-Chain Storage

**Figure 6:**
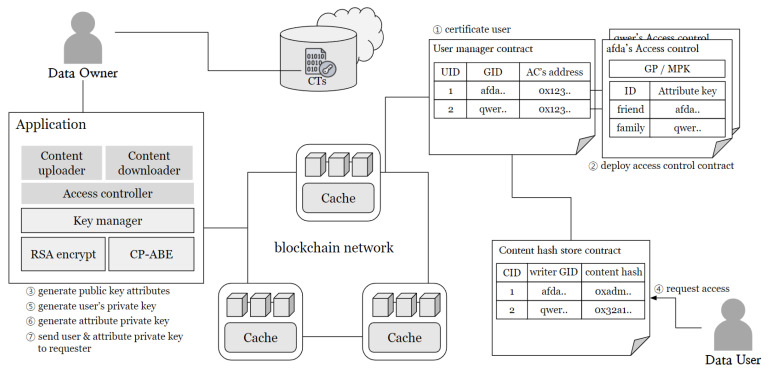
Overview of blockchain-based caching architecture.

This section provides an overview of how distributed access control is performed from off-chain storage, including a description of its key functionalities. To securely store off-chain data, users must encrypt their data, ensuring that only the specified users authorized by the data owner can decrypt it. This section details the methods for granting access to system users. The access control system operates as depicted in Figure 6.

An access controller exists under the content upload and download modules of the application layer. The key manager within this controller performs public key encryption and attribute-based encryption. It generates keys for each encryption, stores the generated keys locally, and performs encryption and decryption functions. Data users encrypt their content through their device’s application before storing it in off-chain storage.

A user management contract is pre-established for system user authentication. The User Manager Contract manages system users, identified through their global IDs, which must be issued by a trusted public certificate authority. This ensures that GIDs are reliable markers of identity. Users authenticate their GIDs using a public–private key pair, ensuring that only those with a verified identity can register. When submitting access requests, users encrypt the GID using their private key. The data owner decrypts this information using the corresponding public key to verify authenticity. If the decrypted GID does not match the claimed GID, then the request is discarded. This mechanism prevents identity spoofing by requiring both the GID and the corresponding private key, ensuring that only legitimate users can access the system.

Each user autonomously deploys their access control contract, registering its address within the user management contract. Through this registered address, users gain access to the public key associated with access control attributes designated by data owners. This mechanism facilitates selective decryption, permitting users to decrypt only the attribute group specified by the respective data owner, thereby enabling flexible utilization across varied contexts. Additionally, users are equipped to transmit access requests to content creators through the content hash store contract. The specific process is as follows:

1. certificateUser(GID): To deploy an access control contract, the user’s GID must be verified as a registered user in the system through $SC_{userManager}$. Registered users can then register their $SC_{accessControl}$.
2. deployContract(GP)→MSK,MPK: The user generates a public–private key pair to control attribute keys through GP and subsequently deploys the access control contract. The contract records the deployer’s MPK and GP and maps the contract address with the user’s GID in SC_userManager_. By managing attribute control through $SC_{accessControl}$, the user can define attributes and control access to them. Users within a specific group can access the contract and perform encryption with the attribute public key, and only the group members with the corresponding attribute keys can decrypt it. This public key management ensures reliable access control, as only the contract deployer can record it.
3. genAttrPubKey(GP)→$SK_{i},PK_{i}$: After contract deployment, the user generates public key pairs for defined attributes. Multiple attributes can be created and used to define access policies. The generated $PK_{i}$ is recorded in the contract, while $SK_{i}$ is stored locally by the attribute creator (DO). Only DO can record valid attribute keys in the contract, ensuring control over attribute management.
4. request(GID): DU requests access by invoking the transaction of the DO’s contract, providing their PK and signed GID to DO. DO verifies the identity of DU and sets access permissions to their data.
5. genUserPrivKey(GID,MSK): Upon detecting a request, DO generates an identification key (K∗_GID_) for DU, preventing unauthorized access through collusion among multiple DUs.
6. $genAttrPrivKey(GP,SK_{i},\mathrm{GID})$→$K_{i},K_{\mathrm{GID}}$: DO assigns attributes to DU and uses the relevant $SK_{i}$ and the requester’s GID to generate user-specific attribute private keys ($K_{i}$) and identification keys (*K*_GID_).
7. $sendKeys(encryptedPK\left(K_{i},K_{\mathrm{GID}},K{*}_{\mathrm{GID}}\right))$: After key generation, DO encrypts the keys with DU’s PK and sends them to DU.
8. encrypt(GP,M,P,{PK},MPK)→CT: DO encrypts the content (*M*) using a set of defined attributes, combining them into a single ciphertext. Only users with attribute private keys matching the policy(*P*) can decrypt the data.
9. $decrypt(GP,CT,P,\left\{K_{i},K_{\mathrm{GID}}\right\},$ *K*${*}_{GID})\to M$: DU attempts to decrypt the ciphertext using their set of attribute keys ($K_{i}$) and $K_{GID}$. If the attribute set matches the access matrix and DU has the correct K∗_GID_, the decryption succeeds.

Through this blockchain-based access control process, user access policies can be managed reliably. CP-ABE encryption ensures that data stored in untrusted off-chain storage is protected while allowing the data owner to control access through a single ciphertext in a distributed environment. Without a centralized key server, the data user directly controls access, preventing data breaches. $N_{cache}$ caches only encrypted content, preventing the risk of content manipulation or unauthorized decryption. Overall, a secure distributed caching system is established through a secure content sharing access control system in a distributed environment.

## 5. Experiments and Evaluation

In this section, we describe the experimental environment for conducting simulations, evaluate the system’s security aspects, and assess the proposed system’s performance to demonstrate its contributions.

### 5.1. Experimental Environment

This section describes the configuration of the experimental environment, including node specifications and parameters.

We evaluate the performance of the proposed system through actual simulations. We adopt Firebase [33] for the off-chain storage layer used for simulation. The blockchain network consists of one conductor node and three physically distinct nodes composing regional cache clusters. Each regional cache node is implemented to form cache cluster with three nodes using Docker v26.1.2, with the regions identified as Korea, UK, and the USA. Thus, the blockchain network comprises a total of ten nodes. To configure the blockchain network, we used geth v1.10.26 to ensure that each cache node forms a private Ethereum [34] blockchain network. For cache cluster configuration, we employed Redis v4.1.3 to set up cache node servers and perform clustering. Smart contracts were written in Solidity and deployed through Truffle v5.11.5. Cache nodes within the blockchain ran the cache manager module to process requests, interact with the cache clusters, and communicate with the blockchain module to interact with the blockchain network. The cache manager module was developed using Python v3.8.10. We used Maxmind [35]’s GeoIP database to obtain the geographic location coordinates corresponding to the IP of the cache node by calculating the distance between user city coordinates based on the city coordinates to which the cache node belongs. The specifications of each node used in the experimental environment are listed in Table 5. We evaluate the system from various aspects by applying various parameters in the simulation. The parameters used in the experiments are shown in Table 6. In order to consider more realistic scenarios, we designed the content request distribution using the Zipf function, allowing us to evaluate the system in terms of its content size, cache size, user request count, cache hit ratio, and off-chain storage aspects.

### 5.2. Security Analysis

In this section, we analyze the security of the system by discussing potential security issues using STRIDE threat modeling. We evaluate how the system addresses or mitigates these issues to ensure robust security.

- **Spoofing**: An attacker could impersonate a user by exploiting the user’s GID and sending authorization requests to the data owner. Both users and cache nodes in the blockchain network register the GID required for signing with the smart contract for identity verification. Authorization requests in the system are signed with the private key of the user’s authenticated GID in the smart contract. Therefore, any request from an attacker who cannot obtain the private key is discarded. Only the user who signed with the private key can modify the blockchain data corresponding to the GID. Thus, each node signs information with the private key corresponding to the GID and communicates with other authenticated users, preventing spoofing attacks by attackers attempting to impersonate other nodes.
- **Tampering:** Because blockchain data cannot be altered, there are two tampering risks in the system. The first is the risk of tampering with data in off-chain storage. An attacker could gain access to off-chain storage and modify the data. The proposed system prevents tampering by verifying the integrity of the data retrieved from off-chain storage. The hash of the original data is recorded on the blockchain, so the integrity is verified by comparing the requested data with the blockchain data. The second risk is that cache nodes could arbitrarily tamper with cached data. This tampering can also be detected through user verification post-request, and malicious cache nodes can be removed to mitigate the risk. Therefore, the system ensures the trustworthiness of nodes and the integrity of the data, preventing unauthorized data modifications.
- **Repudiation:** An attacker could undermine the system’s reliability by claiming that updated content was not actually updated. The system requires transactions to be signed with a private key to modify blockchain values through the smart contract. Because blockchain values must be updated during the update process, users cannot deny that they performed the update if it was successful. Only the user who signed the transaction can perform records on the smart contract, so all information regarding content registration and updates is recorded and verifiable on the ledger. Additionally, a malicious cache node could claim that it did not receive a request or send manipulated data. If the cache node does not respond within a certain time frame or sends incorrect content, its authority, recorded in the smart contract, can be revoked; thus, the system’s security is ensured by blocking malicious cache nodes.
- **Information Disclosure:** An attacker could exploit the public ledger recorded on the blockchain to obtain and manipulate user information. However, the main information recorded on the blockchain in the system is the user’s global ID and the hash value of the content, making it impossible to attack system users based solely on the disclosed information. Additionally, an attacker could attack off-chain storage and cache nodes where the original data are stored in order to leak the stored data. However, the original content is encrypted based on attributes, and only users with the attribute key can decrypt it. The attribute key is also issued directly by the data user and encrypted with the requester’s public key, meaning that no one other than the involved parties can obtain the key. Therefore, security regarding leaks is enhanced, as the data owner directly manages access rights.
- **Denial of Service:** An attacker could paralyze the system by discovering and attacking the information of the conductor that configures the cache cluster. However, because the conductor is randomly selected from the cache nodes in the system, it is difficult to attack. Additionally, cache nodes continuously check the conductor’s status, and the conductor also continuously monitors the status of the cache nodes, allowing continuous management of the cluster’s state. Furthermore, cache nodes can verify and reject unauthorized users within the system. In order for an attacker to paralyze the entire cache cluster, they would need to gather information on and attack all distributed cache nodes in the blockchain network, which is difficult to execute.
- **Elevation of Privilege:** Data owners can directly grant data access rights to users based on specified attributes. Because data owners directly deliver the attribute key to the users, attackers cannot obtain the rights to the data. However, an attacker with attribute A and another attacker with attribute B in the system might attempt to combine their attribute keys to decrypt content encrypted with an “A AND B” attribute policy. However, since different identification keys are generated for attributes per user, if the two users are differently identified, they cannot satisfy the attribute policy. Therefore, colluding attacks that attempt to satisfy access policies are impossible. Only authenticated users can request authorization, and the owner directly approves it, preventing privilege escalation attacks.

### 5.3. Performance Evaluation and Analysis

This section approaches the analysis of system performance using various parameters.

**Figure 7:**
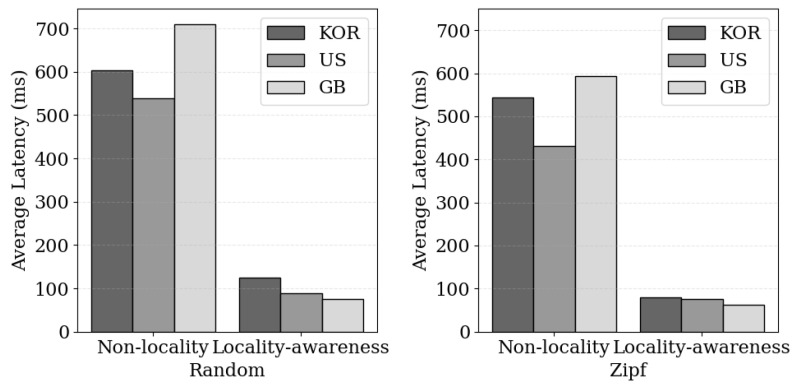
Comparison of request processing latency based on request patterns and locality-aware cache cluster configuration; (Random) indicates that the requested content is randomly selected and requested, while (Zipf) is a method for randomly changing the requested content according to its priority.

When forming cache clusters with blockchain nodes distributed globally, without considering locality, the distance between the cache nodes caching user data and the users themselves may be physically distant. Figure 7 illustrates the comparison of average request processing latency between a scenario where blockchain nodes are distributed globally and one where cache clusters are formed based on locality. The experiments were divided into two scenarios; the first involved randomly requesting one of the 1000 stored contents, while the second considered a Zipf distribution based on content popularity to send requests, with 5000 requests sent to measure the average latency. The average size of the content was 64 KB. In the case where locality was not considered, network delays occur due to cache nodes based on globally distributed blockchain nodes. In contrast, when considering locality, clients send requests to cache nodes in their closest region, resulting in similar or improved latency, demonstrating the efficiency of regional cache clustering. This was effective in both the random and Zipf scenarios, showing fast request processing performance of 95 ms in the Zipf scenario, which reflects realistic biased content popularity situations. These experimental results demonstrate that performance optimization through cache clustering when considering locality in the blockchain network is achievable.

**Figure 8:**
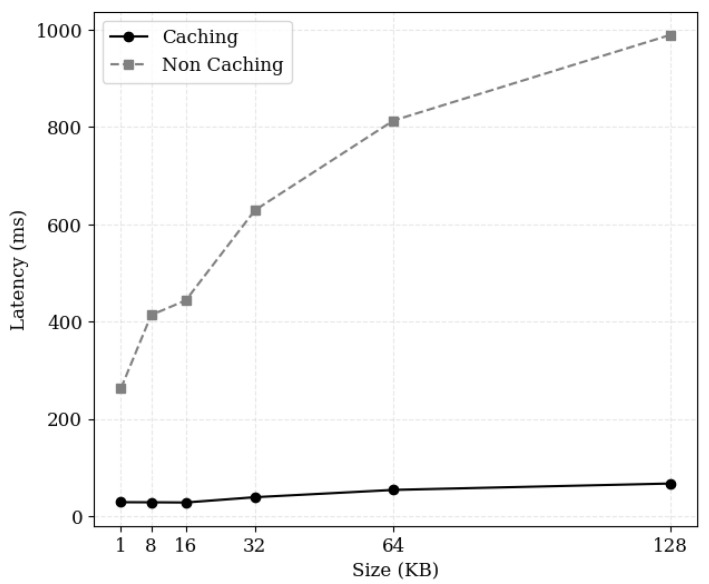
Request processing latency by content size based on caching status; (Caching) indicates that the requested content is cached, while (Non Caching) indicates that the requested content is stored only in external storage.

Figure 8 illustrates the average request latency according to the requested content size. It shows the difference in latency between requesting uncached content and cached content. When content is uncached, the measured latency represents the time taken to fetch data directly from cloud storage; when content is cached, it represents the time taken to fetch data directly from the cache cluster. For 1 KB of data, the average latency when hitting the cache was 28 ms, whereas fetching directly from the cloud resulted in an average latency of 262 ms, a difference of more than nine times. On the other hand, for 128 KB data, the average latency when hitting the cache was 67 ms, while downloading directly from the cloud took 989 ms, a difference of over fifteen times. This demonstrates that applying caching mechanisms results in a latency reduction of over 89%, proving particularly effective in large-scale data transfers.

**Figure 9:**
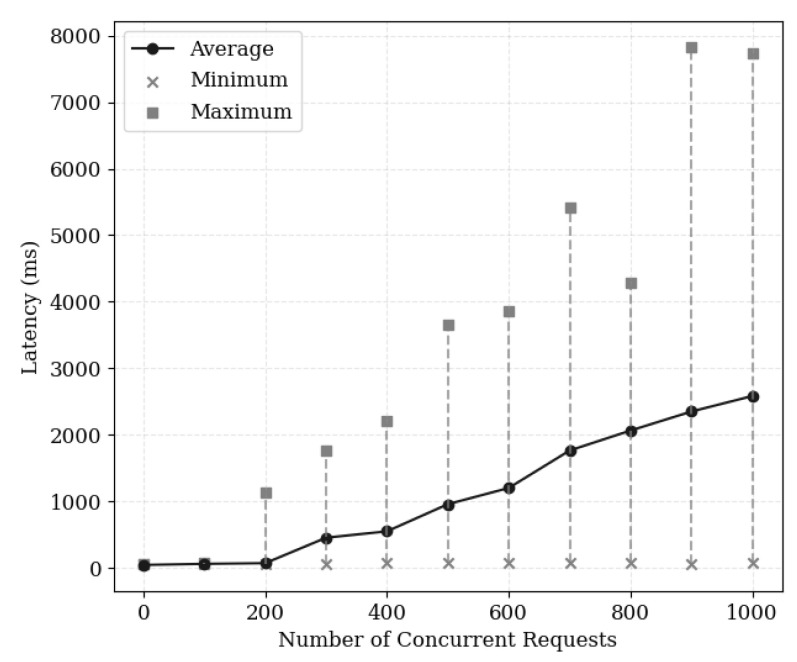
Request processing latency based on number of concurrent users.

Figure 9 illustrates the latency for concurrent user requests on cached data. The average latency remains relatively stable up to an initial 300 concurrent requests, after which it sharply increases. While the minimum latency values remain relatively stable, the maximum latency values fluctuate greatly, and increase sharply with higher concurrent request numbers. When measuring the latency for requests to a single cache node, we observed bottlenecks in the load both of that node and of the network bandwidth, leading to a sharp increase in latency at specific intervals. However, In a distributed environment with multiple cache nodes, increasing the number of cache nodes can provide faster latency to a larger number of users. Consequently, there is a need to establish optimization strategies for cache node specifications and quantities according to user scale.

**Figure 10:**
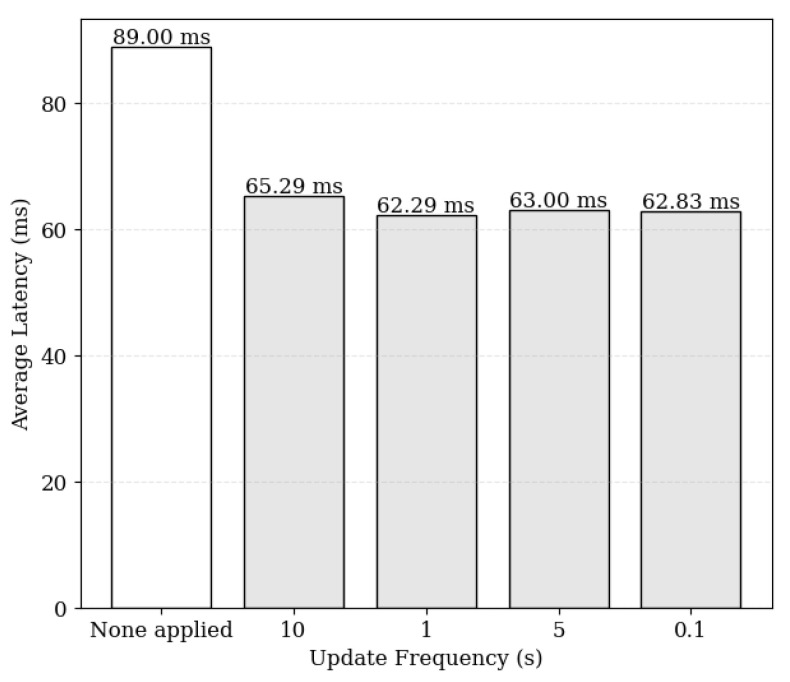
Request processing latency for different update frequencies.

The proposed system utilizes the smart contract’s update event logs to validate the consistency of data cached by each cache node. By defining update events and listening to them, cache nodes can track updates and verify the consistency of their cached data for delivery to users. Figure 10 illustrates the results of measuring response latency based on the frequency of event occurrences. Without leveraging event logs, the process of comparing blockchain data with caching data for consistency verification resulted in a latency of 89 ms per request. However, when processing requests by detecting update events defined in the smart contract, the maximum latency was reduced to 65 ms. Despite frequent updates, the system demonstrates minimal performance degradation or impact on responsiveness. These findings indicate a 26% reduction in latency compared to reading local ledger data, ensuring consistency with relatively low overhead.

**Figure 11:**
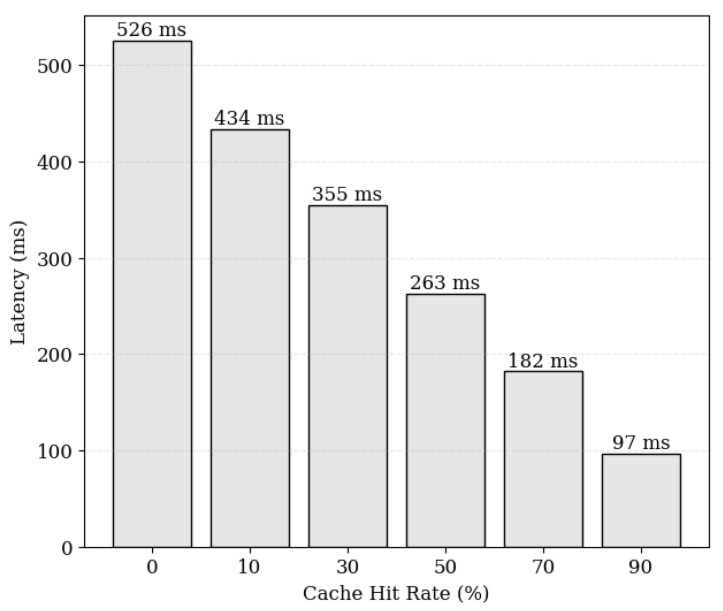
Average response latency based on cache hit ratio.

**Figure 12:**
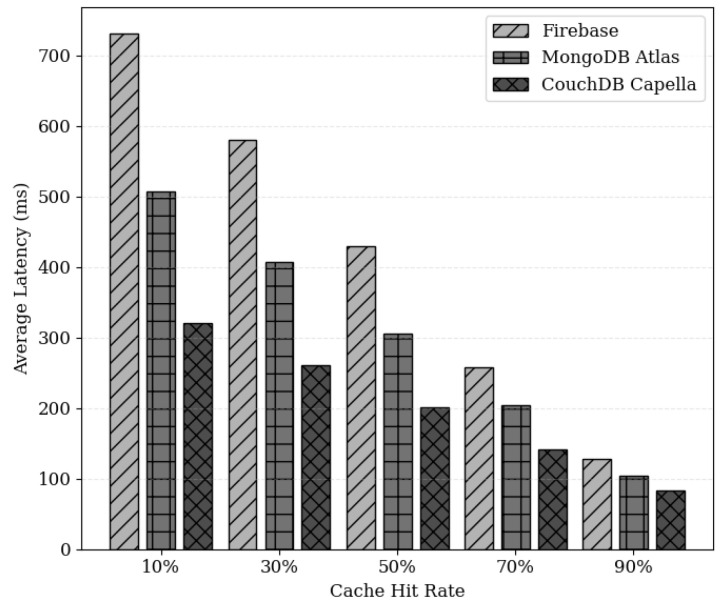
Response latency based on cache hit ratio for off-chain storage.

Figure 11 illustrates the relationship between cache hit ratio and average latency, demonstrating that the data retrieval time decreases as the cache hit ratio increases, resulting in reduced response latency. With a higher cache hit ratio, a maximum reduction in latency of up to 81% is observed. Hence, it is imperative to consider policies that can effectively increase cache hit ratios. Figure 12 illustrates the latency across different off-chain storage layers based on the cache hit ratio. Firebase, MongoDB Atlas, and CouchDB Cappella were utilized as off-chain storage layers, all of which are located in the United States. The results indicate that CouchDB Cappella exhibits the lowest average latency; across all cloud storage options, the average latency decreases as the hit ratio increases. This suggests that applying the caching system on any cloud storage layer can yield performance benefits. Additionally, the experimental results underscore the importance of optimizing system performance by selecting an appropriate storage solution while considering the performance changes with varying cache hit ratios.

**Figure 13:**
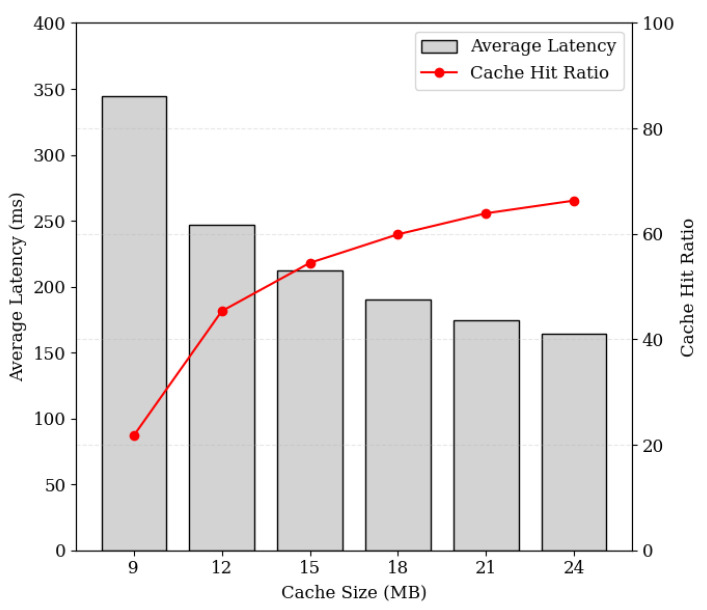
Average response latency and cache hit ratio based on request and cache data replacement simulation.

In addition, to validate the effectiveness of the system it is necessary to apply the appropriate cache replacement policy in real-world constrained environments. Figure 13 illustrates the cache hit ratio and latency when employing the Least Recently Used (LRU) replacement policy in cache replacement processes while considering limited cache sizes for each cache cluster. The experiment comprises 5000 requests, with request patterns defined using Zipf distribution for realistic simulation. Each content has a size of 64 KB, totaling 64 MB of content in cloud storage. The results demonstrate a gradual decrease in average latency with increasing cache size. For instance, with a cache size of 9 MB (approximately 14% of the total storage size) the cache hit ratio is around 21%, while for a cache size of 21 MB (approximately 32% of the total storage size) the hit ratio increases to 64%. These findings highlight the efficacy of the cache system in resource-constrained cache clusters. By appropriately adjusting the cache size, it becomes feasible to achieve high cache hit ratios and low response latency. Thus, the experiment provides empirical evidence for determining the optimal cache size. Furthermore, leveraging cache replacement policies that enhance cache hit ratios can further maximize performance improvements.

**Figure 14:**
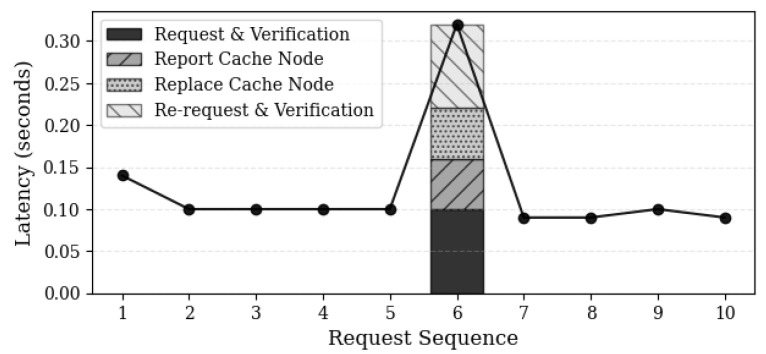
Time required for issue detection and cache node replacement to occur. After attempting to cause a failure in the sixth request sequence, the failure recovery time and the proportion of each process delay are measured.

Figure 14 illustrates the process of detecting intentional delivery of corrupted content by cache nodes that directly provide content and selecting new cache nodes. In the sixth request sequence, a cache node intentionally delivers corrupted content. Upon receiving the corrupted content, users verify the integrity of the content to determine whether the cache node delivered the correct content. If corruption is detected, users report the cache node to the cache manager contract. Consequently, the cache manager contract records the malicious cache node in the list of malicious cache nodes within the blockchain, preventing it from further cache node registrations. After reporting, the users request a new connection from the conductor and select a new cache node according to the existing process. Subsequently, the selected cache node receives cached data from the actual cluster, verifies data integrity, and receives the correct data. Typically, there is a delay of 0.1 s between receiving and verifying the requested data. The process of removing a malicious cache node and recovering the cache node incurs an additional delay of approximately 0.22 s, indicating that the overhead required for system recovery is not significant.

The overarching goal of the proposed system is to enhance the performance of existing blockchain-based off-chain storage solutions. To assess the potential improvement in performance compared to previous research, a comparative analysis was conducted. The study extracted retrieval latency times from the literature for systems utilizing IPFS as off-chain storage; the retrieval latency of the system using cloud storage was directly measured using Firebase as the cloud storage, mimicking an environment in which only the caching system of the proposed system is removed. Table 7 presents a comparison of latency times across each system. The proposed system demonstrated a latency of 0.067 s under 100% cache hit rate conditions and 0.526 s when the cache hit rate was 10%. Even with a lower hit rate setting, the proposed system showed a latency difference more than twice that in the literature. This confirms that the proposed system effectively mitigates performance overhead for off-chain storage that has not been accessed previously.

Collectively, by considering factors such as locality in cache clustering, leveraging smart contract update events, and optimizing cache hit ratios, the proposed system demonstrates substantial reductions in latency along with improved performance. Indeed, the combination of performance enhancements with the implementation of robust data integrity validation mechanisms holds the potential to significantly bolster the efficiency and reliability of caching mechanisms within blockchain-based applications. By leveraging these improvements, blockchain applications can effectively mitigate latency issues, enhance responsiveness, and ensure the consistency and reliability of data storage and retrieval processes.

## 6. Conclusions

The proposed system introduces a novel approach for enhancing the performance of DApps by integrating a caching architecture into existing off-chain storage solutions, thereby ensuring the integrity and secure management of application data. Through the cache manager, the system constructs regional cache clusters based on blockchain nodes to deliver fast request processing latency to users. Additionally, leveraging attribute-based encryption protects data stored in untrusted cloud storage from leakage and unauthorized decryption enables access control for multiple users with a single ciphertext. The proposed system underwent a security evaluation and performance comparison simulations. The results confirm a significant improvement in request processing speed, with a reduction of over 89% compared to the pre-application system in terms of regional cache clustering and cache consistency. Considering the direct correlation between cache hit ratio and system performance, future research should focus on securing cache storage resources and developing cache replacement policies to maximize performance.

Ultimately, the proposed system ensures the secure storage and transmission of user content in distributed environments while overcoming the performance issues inherent in existing decentralized applications. This design can be applied across various domains operating in the Web3.0 environment, potentially fulfilling the role of CDNs (Content Delivery Networks) in improving request performance for centralized servers. Consequently, it fosters a decentralized application environment that guarantees real-time responsiveness, providing users with a seamless experience.

## Figures and Tables

**Table 1:** Comparison of caching research for distributed environments.

	Abstract	Contribution	Differences
Ymanaka et al. [15]	A user-centric in-network caching mechanism for ICN-based off-chain storage to address the limitations of existing off-chain solutions.	Caches data based on user location to shorten response time. Utilizes ICN routing protocols for efficient data transmission between network nodes.	Presence of a key server managing keys in a distributed environment poses security threats due to centralization. ICN is in its early stages, making it difficult to apply complex structures and processes.
Ymanaka et al. [15]	A distributed and proactive caching strategy based on blockchain for mobile edge computing environments.	Introduces a proactive caching strategy based on node utility to optimize caching. Addresses the trade-off between blockchain consensus process delay and content caching delay. Achieves optimal caching strategy using linear relaxation techniques and internal point methods.	Delay in cache transaction process through blockchain between users and cache nodes. Oracle issues arise due to offline proofs between data providers and cache nodes, leading to decreased system reliability.
Bai et al. [16]	Propose a distributed and proactive caching strategy based on blockchain for mobile edge computing environments.	Introduces a proactive caching strategy based on node utility to optimize caching. Addresses the trade-off between blockchain consensus process delay and content caching delay. Achieves optimal caching strategy using linear relaxation techniques and internal point methods.	Delay in cache transaction process through blockchain between users and cache nodes. Oracle issues arise due to offline proofs between data providers and cache nodes, leading to decreased system reliability.
Guo et al. [17]	Improves data security and caching hit rate in edge computing environments using blockchain technology.	Ensures data security in edge environments using blockchain. Improves search performance through caching optimization models.	Specifically designed for edge environments. Delays occur in IPFS search and backup processes between edge nodes and blockchain nodes.
Zhang et al. [18]	A three-layer caching mechanism including cache node layers in large-scale distributed networks.	Provides faster response to users through hotness-based dynamic caching. Converts large files into Merkle tree structures and stores them using IPFS systems after encryption.	Relies on a TPA (Third-Party Auditor) for data integrity verification, posing issues around data reliability and single points of failure.
Heo et al. [19]	A blockchain-based decentralized framework for proactive caching in hierarchical wireless networks.	Combines blockchain and game-theoretic perspectives to design a distributed caching system. Provides approaches to activate cache helpers through incentive mechanisms.	Lacks a method for ensuring the integrity and consistency of stored cache data.
Wang et al. [20]	Proposes MLDC multi-level distributed caching strategy for blockchain storage optimization.	Reduces storage requirements by storing only necessary data instead of full data replication. Enables data integrity verification and requesting of necessary data using hash values.	Increase in network traffic when requesting data from other class nodes. Difficulty in guaranteeing minimum nodes occupying the upper layer among all nodes, leading to system instability.
Freedman et al. [21]	Proposes CoralCDN, a web content distribution network utilizing bandwidth from volunteer resources.	Minimizes hotspot congestion through two-stage insertion algorithms. Improves content latency using hierarchical cluster systems.	Delays occur due to use of proxy and DNS servers for requests between clients and Coral clusters, leading to delays in inter-cluster exploration.
Saturn [22]	Proposes blockchain-based L1 nodes for caching IPFS-stored data.	Implements a content delivery network consisting of L1 nodes to accelerate content delivery. Encourages L1 node availability through incentive mechanisms.	Lacks universality due to supporting only IPFS-based content delivery networks.

**Table 2:** Comparison of related research on blockchain-based systems.

	Decentralized	Cache System	Preventing Cache Poisoning	Data Integrity	Access Control
[15]	low	✓	×	✓	✓
[16]	middle	✓	×	✓	×
[17]	high	✓	×	✓	×
[18]	high	✓	✓	✓	×
[20]	middle	✓	✓	×	×
[24]	middle	×	×	✓	✓
[25]	middle	×	×	×	✓
[26]	middle	×	×	×	✓
[27]	middle	✓	×	✓	✓
[28]	middle	×	×	✓	✓
Proposed	high	✓	✓	✓	✓

**Table 3:** CP-ABE function descriptions.

Function	Description
initCircuit(λ)→GP	Circuit initialization
configCA(GP)→(SK∗,PK∗)	Certification Authority configuring
$genUserPrivKey\left(GP,RL,GID,SK*\right)\to K{*}_{\mathrm{GID}}$	Private key generation for user certification
genAttrKey(GP)→(SK,PK)	Attribute key pair generation
$genAttrPrivKey\left(GP,SK,GID,i\right)\to K_{i},K_{\mathrm{GID}}$	Attribute private key generation for data user
$encrypt(GP,M,P,\left\{PK\right\},PK^{*},RL)\to CT$	Message encryption to generate ciphertext
$decrypt(GP,CT,P,\left\{K_{i},K_{\mathrm{GID}}\right\},K{*}_{\mathrm{GID}},RL)\to M$	Ciphertext decryption to recover the original message

**Table 4:** Frequently used notation.

Notation	Description
BCN	Blockchain network
SC	Smart contract
*U*	System user
*N*	Node in blockchain network
$N_{cache}$	Cache node in blockchain network
$N_{cdt}$	Conductor node for cache cluster
DO	Data owner of system
DU	Data user of system
PK	User’s public Key
SK	User’s private Key
MPK	Data owner’s master public Key
MSK	Data owner’s master private Key
K∗	User identification private key
$K_{i}$	private key for attribute *i*

**Table 5:** Experimental environment.

Type	Location	Specifications
**Conductor**	KR	Window 11, CPU Intel Core i9-13900KF, RAM 64 GB, SSD 1 TB
**Cache node**	KR	Ubuntu 20.04, vCPU 2 core, RAM 4 GB, SSD 50 GB
	US	Ubuntu 18.04, vCPU 2 core, RAM 4 GB, SSD 50 GB
	UK	Ubuntu 22.04, vCPU 1 core, RAM 1 GB, SSD 30 GB
**Client**	KR	Ubuntu 22.04, vCPU 1 core, RAM 1 GB, SSD 30 GB
	US	Ubuntu 22.04, vCPU 1 core, RAM 1 GB, SSD 30 GB
	UK	Ubuntu 22.04, vCPU 1 core, RAM 1 GB, SSD 30 GB

**Table 6:** Experiment parameters.

Parameter	Value
Zipf factor	1
Content size	1–128 KB
Cache size	9–24 MB
Number of users	1–1000
Total number of contents	1000
Number of caching contents	100–900

**Table 7:** Performance comparison of research on blockchain-based systems; request file size = 100 KB.

System	Retrieval Latency (s)
Blockchain based system with IPFS [13]	3
Blockchain based system with cloud storage [14]	1.155
Proposed system	0.067–0.526

## Data Availability

The original contributions presented in the study are included in the article material, further inquiries can be directed to the corresponding author.
